# An Unusual Case of Gynecomastia Associated With Subclinical Hyperthyroidism

**DOI:** 10.7759/cureus.51969

**Published:** 2024-01-09

**Authors:** Pedro L Rojas, Rumeysa Ucar, Lutfor Nessa, Maria C Perez, Devi Suravajjala

**Affiliations:** 1 Internal Medicine, Texas Tech University Health Sciences Center, Odessa, USA; 2 Medical School, University of Pamplona, Cúcuta, COL; 3 Endocrinology, Diabetes and Metabolism, Texas Tech University Health Sciences Center, Odessa, USA

**Keywords:** thyroiditis, subclinical, male, gynecomastia, hyperthyroidism

## Abstract

Gynecomastia in males is a medical condition that manifests as the abnormal enlargement of male breast tissue and has a variety of potential causes, which mainly classify as physiologic (infancy, puberty, elderly) and pathologic (hyperthyroidism, medications, cirrhosis, chronic kidney disease (CKD), malignancies). Pathologic causes mainly result from hormonal imbalances. While gynecomastia has been documented in cases of Graves’ disease, it is rarely the presenting symptom with very few cases reported in the literature. Here we report an uncommon case of a 65-year-old male with bilateral gynecomastia who presented to his primary care physician (PCP) with concern for breast sensitivity and enlargement. Ultrasound of his breasts showed bilateral findings consistent with gynecomastia. Initial lab demonstrated suppressed thyroid-stimulating hormone (TSH) levels high, normal FT4, elevated estradiol level, elevated sex hormone-binding globulin (SHBG), and elevated total testosterone. The patient was seen by an Endocrinologist six months post-symptom onset and reported that his symptoms had resolved spontaneously.

## Introduction

Gynecomastia in males is a medical condition that manifests as the abnormal enlargement of male breast tissue and has a variety of potential causes. True gynecomastia is a common feature often related to estrogen excess and/or androgen deficiency as a consequence of hormonal imbalances [1-3]. Hyperthyroidism may be associated with or rarely can be presented with gynecomastia as an initial and only manifestation [4]. Gynecomastia, as a feature of thyrotoxicosis, is seen in 10-40% of cases [3].

The underlying mechanism is not completely clear. It is hypothesized that the imbalance between free testosterone and estrogen-induced by thyroid hormones may result in increased production of hepatic sex hormone-binding globulin (SHBG), potentially contributing to the development of gynecomastia in hyperthyroidism [3,5-8]. SHBG has a higher affinity to binding testosterone, which results in a decreased level of free testosterone [2-5]. Thyroid hormones stimulate aromatase which converts androgen into estrogen in peripheral tissue [1,5-8].

Hyperthyroidism-induced gynecomastia can be managed by achieving an euthyroid state. The first-line treatment is the use of antithyroid medications. Generally, gynecomastia tends to resolve after a few months of treatment.

## Case presentation

A 65-year-old male with no significant medical history presented to the primary care physician (PCP) on 10/25/22, with the main concern for progressive breast sensitivity and enlargement for two months, associated with weight loss but the exact specifics of weight changes were not known to the patient. These symptoms were noted during the time the patient injured his right hip after a ground-level fall on 08/30/22 before he underwent right hip hemiarthroplasty on 09/02/22. He had no other medications at home including over-the-counter (OTC) supplements. He denied any changes in libido, morning erections, or shaving frequency. Other reviews of systems were negative including autoimmune conditions, liver or kidney stigmata, alcohol use, chronic opioid use, or any other drug use. Vitals signs were unremarkable. At the time of symptom onset, his calculated BMI was 18 kg/m^2^. Physical examination of the breast revealed the presence of sensitive bilateral crusty nipples with palpable nodules of about 4 cm with no discharge. The rest of the physical exam was normal, including testicular exam.

Ultrasound of breasts done on 11/16/22 showed bilateral benign-appearing masses in the subareolar region with no pathologic calcifications or architectural distortion consistent with bilateral gynecomastia, no specific size measure was reported on ultrasound. The mammogram was difficult to perform due to the patient’s lean body habitus. Initial evaluation before the right hemiarthroplasty as shown in Table 1 revealed suppressed thyroid-stimulating hormone (TSH) levels and elevated free thyroxine (FT4). The patient was not placed on any therapy. After two months, further workup with his PCP again demonstrated suppressed TSH levels and high normal FT4. A thyroid function panel was ordered by PCP due to the patient’s history of subclinical hyperthyroidism and to evaluate this as the underlying etiology of his gynecomastia. Lab results also showed elevated estradiol level, elevated SHBG, elevated total testosterone, normal free testosterone, normal human chorionic gonadotropin (hCG) beta-subunit, and normal prolactin level (Table 1).

**Table 1:** Laboratory findings of the patient over time. Free T4: free thyroxine; Total T3: total triiodothyronine; TSH: thyroid stimulating hormone; SHBG: sex hormone-binding globulin; LH: luteinizing hormone; TSI: thyroid-stimulating immunoglobulin; ALT: alanine aminotransferase; AST: aspartate aminotransferase; hCG beta subunit: human chorionic gonadotropin beta subunit

Laboratories	Reference range	At the time of surgery	Initial visit	After four months
Free T4 (ng/dL)	0.82-1.77	3.97	1.75	1.28
Total T3 (ng/dL)	80.0-200.0	-	-	72.3
TSH (uIU/mL)	0.450-4.500	<0.015	<0.005	0.049
SHBG (nmol/L)	19.3-76.4	-	103	65.1
Free testosterone (pg/mL/ng/dL)	34.7-150.3/4.1-23.9	-	108.6	8.92
Total testosterone (ng/dL)	264-916	-	1,090	682
LH (mIU/mL)	1.7-8.6	-	8.1	-
TSI (IU/L)	<=0.54	-	-	0.41
Prolactin (ng/mL)	4.0-15.2	-	10.9	-
Creatinine (mg/dL)	0.76-1.27	0.5	0.64	-
ALT (IU/L)	0-44	23	16	-
AST (IU/L)	0-40	29	22	-
hCG beta subunit (mIU/mL)	0-3	-	<1	-
Estradiol (pg/mL)	7.6-42.6	-	46.5	39.3

Following this, the patient was referred to our endocrinology department. The patient was seen by an Endocrinologist six months post-symptom onset and reported that his symptoms had resolved spontaneously. Physical exam including breasts and thyroid was normal. By this time his BMI had improved to 28, over the last six months.

Repeated labs showed a persistent suppressed TSH but improved relative to the previous level, a normal FT4, and negative thyroid-stimulating immunoglobulin (TSI). Levels of total testosterone, SHBG, and estradiol reverted to normal. All initial and follow-up labs are shown in Table 1.

Although TSH remained suppressed on follow-up labs, it improved compared to the previous value, along with the asymptomatic state of the patient and a negative TSI as mentioned; a diagnosis of transient hyperthyroidism was made. However, the radioactive uptake scan and thyroid ultrasound could not be completed due to patient transportation issues. As the patient was asymptomatic, no further treatment was needed. The patient reported difficulties in following up with different physician’s appointments, as he lives alone and has transportation issues. He was instructed to follow up with his PCP for repeat thyroid labs in three months and to come back to the Endocrinology clinic if requested by PCP. Unfortunately, he was lost to follow-up.

## Discussion

Gynecomastia in males has rarely been reported as the presenting symptom of hyperthyroidism over more than 60 years of reporting on thyrotoxicosis (10-40% cases of thyrotoxicosis) [2-4]. Previous studies showed no correlation between the presentation of gynecomastia and the severity of hyperthyroidism [5]. There are few cases of gynecomastia in men in the literature with Graves’ disease. As far as we know, no other cases were reported with the initial complaint of gynecomastia in patients suffering from transient hyperthyroidism, as seen in our patient.

There are several reasons why gynecomastia could be identified as the initial complaint in a hyperthyroid state. It may be the primary symptom, a significant symptom, or one among multiple symptoms that the patient or physician does not initially identify [6,7].

Even though there is a lot of research in the literature about the pathophysiology of gynecomastia in hyperthyroidism, the underlying mechanism is not completely clear [1,5,6]. It is hypothesized that two mechanisms may be responsible for developing gynecomastia in hyperthyroidism. The first mechanism is the imbalance between free testosterone and estrogen induced by thyroid hormones may result in increased production of hepatic SHBG [3,6-8]. SHBG has a higher affinity to binding testosterone, which results in a decreased level of free testosterone, the active form of testosterone. Decreased free androgen reduces negative feedback control in the pituitary and stimulates luteinizing hormone (LH) production [2,5]. LH provokes the Leydig cells to increase the production of androgen and estradiol (E2). This mechanism helps to normalize free androgen but results in elevated free estrogen and increases the estrogen-testosterone ratio [1,5,7]. Disproportionate estrogen will further promote SHBG and alter the active E2 and T ratio [6,7].

The second mechanism is an increased level of aromatase activity. Thyroid hormones stimulate aromatase which converts androgen into estrogen in peripheral tissue. Increased levels of LH also prompt aromatase activity. This hormonal imbalance between estrogen and testosterone results in gynecomastia [1,5]. Elevated E2 level in a patient with hyperthyroidism does not necessarily result in gynecomastia. The duration of exposure to high levels of E2 hormone, target tissue responsiveness, and level of aromatase activity also affects the presentation of symptoms of gynecomastia. In our case, lab tests revealed elevated levels of SHBG, total testosterone, estradiol, and a normal range of free testosterone and LH [5,7]. Some patients may also have “subclinical” gynecomastia [7]. A previous histological study by Becker et al. revealed evidence of gynecomastia in 15 (83%) out of 18 hyperthyroid men who underwent bilateral breast biopsies at the time of subtotal thyroidectomy even though breast enlargement was recorded clinically in six patients (33%) [7,9]. We are not sure whether the transient hyperthyroid state in our case could be related to the stressor of the patient’s recent right hip hemiarthroplasty. Overall, limited data indicates that psychological stress may contribute to the etiology of hyperthyroidism, probably along with other predisposing and/or precipitating factors [10]. Although, this is less likely as our patient’s TSH was already suppressed before the surgery. Another differential could have been subacute thyroiditis, but our patient lacks the most common symptoms such as fever and neck pain [11]. Also, painless thyroiditis presents with a similar picture characterized by transient thyrotoxicosis caused by the destruction of the thyroid gland, resulting in excess levels of thyroid hormone. This thyrotoxicosis improves spontaneously and is often followed by a hypothyroid phase and complete recovery [12]. These last conditions seem less likely as the patient’s TSH level remained suppressed even after months. One major limitation in our case is that the patient was lost to follow-up, so no further workup was done to look for the exact etiology of his transient hyperthyroidism.

Treatment of hyperthyroid-induced gynecomastia is to achieve a euthyroid state. This could be achieved with antithyroid medications, radioiodine treatment, or thyroidectomy [13]. Previous case reports also revealed that gynecomastia was determined even after euthyroid state restoration [3,5,7]. In this case, repeated levels of SHBG, E2, and total T were back to normal with no treatment initiation that correlated with mastalgia and gynecomastia resolution around six months after the onset, precisely two weeks before his visit to the endocrinology clinic. The patient's TSH also improved from <0.005 to 0.049 with normalization of free T4 and total T3.

## Conclusions

Gynecomastia can be the initial clinical manifestation of thyroid hormone abnormalities. Our case highlights a rare presentation of gynecomastia with transient hyperthyroidism. Clinical suspicion is vital to assess thyroid hormone levels in all men with gynecomastia. This will help reduce unnecessary interventions as hyperthyroidism-induced gynecomastia is sensitive to the euthyroid state and as with our patient, sometimes treatment is not necessary.
